# Antimicrobial Activity of Plasma Rich in Platelets (PRP) on the Ocular Microbiota of Healthy Horses from Minas Gerais: *In Vitro* Study

**DOI:** 10.1155/2023/2407768

**Published:** 2023-08-16

**Authors:** Luis E. C. Torres, Camilo O. Florez, Jéssica G. Oliveira, Giovanna D. Vieira, Ilza S. Ribeiro, Kelly M. Keller, Fabíola O. P. Leme, Priscila Fantini, Renata P. A. Maranhão

**Affiliations:** ^1^Escola de Veterinária, Universidade Federal de Minas Gerais, Belo Horizonte, Minas Gerais 31270-901, Brazil; ^2^Grupo UnieduK, Centro Universitário de Jaguariúna-UNIFAJ, Jaguariúna, São Paulo 13918-110, Brazil

## Abstract

In equine ophthalmology, ulcerative keratitis is among the most common conditions and, in general, arises as a consequence of some trauma suffered. Secondarily, subsequent contamination by pathogenic or resident bacteria of the horse's ocular microbiota may have undesirable consequences. Under physiological conditions, the normal microbiota coexists with the immune status of the host, serving as a barrier, ensuring the health of the ocular surface, and inhibiting the proliferation of pathogens. However, in the imbalance of immune barriers, the normal microbiota can become pathogenic and lead to infection, acting as an opportunistic agent. The present study aims to demonstrate the antimicrobial effect of platelet-rich plasma (PRP), its time of action, and its correlation with the concentration of its same components *in vitro* on *Staphylococcus sciuri*, a bacterium with high prevalence in the normal ocular microbiota of horses in the municipality of Minas Gerais. For the preparation of the PRP, eight adult Quarter Horse (QH) horses were used. The individual PRP was prepared by the double centrifugation protocol, and then, the PRPs were added to a pool, followed by testing their interaction in culture with Brain Heart Infusion (BHI) broth at different dilutions against five strains collected from different animals. After 3, 6, 12, and 18 hours, the colony formation units (CFU) count on a 5% horse blood agar plate was evaluated for each time point. Our study showed that *Staphylococcus sciuri*, the resident microorganism of the ocular conjunctival microbiota of horses, is more susceptible when compared to the standard strain “American Type Culture Collection” (ATCC-29213) *Staphylococcus aureus*, a pathogenic microorganism, which was used for the validation of our study. The antibacterial effect shown in this study was bacteriostatic for up to 6 hours. The most concentrated PRP dilutions, 1 : 1 and 1 : 2, were also most effective, suggesting that the antibacterial effect is volume dependent.

## 1. Introduction

Regenerative medicine and tissue engineering are rapidly growing fields that aim to restore or replace damaged or diseased tissues using a variety of approaches. One promising approach is the use of platelet-rich plasma (PRP), a concentrate of platelets obtained from the patient's own blood. PRP contains various growth factors and cytokines that can enhance the healing and regeneration of tissues.

Several studies have investigated the use of PRP in regenerative medicine and tissue engineering in both humans and animals. The PRP has a positive impact on tissue regeneration particularly in bone and soft tissue healing [1]. PRP is found to stimulate cellular growth and differentiation, increase collagen synthesis, and enhance angiogenesis [2]. It can also help reduce inflammation and pain associated with tissue damage [3].

The use of platelet concentrates has gained significant attention in recent years as a potential treatment for wound healing. Clinical studies show that these platelet concentrates are effective treatments for a variety of wounds, both in humans and animals, including chronic ulcers, burns, and surgical incisions [1, 4, 5].

Platelet concentrates have been used in a variety of clinical settings, including orthopedics [6, 7], dentistry [8, 9], and ophthalmology [10] to enhance tissue repair and regeneration. In veterinary medicine, PRP has been used to treat various conditions in animals. The authors in [5] investigated the use of PRP in dogs with chronic skin wounds. The study found that PRP treatment can improve wound healing and reduce the risk of infection. This shows that in addition to its regenerative properties, PRP has antibacterial properties, which have already been proven in various experimental models with animals [11–13].

Ulcerative keratitis is among the most common conditions in equine ophthalmology [14]. Although trauma is the primary cause, secondary resident or pathogenic bacterial contamination might result in severe sequelae.

The bacterial isolates from the normal conjunctiva may vary depending on the geographic location, climate, age, sex, and habitat of the animals. Most commonly, aerobic Gram-positive bacteria predominate [19–21]. The *Staphylococcus* genus is found in several studies, always being the 1st or 2nd most frequent genus among studies and with occurrences above 18% [17, 19–21]. Thus, it is possible that *Staphylococcus* spp. is the only truly resident microorganism and that the others are transient microorganisms, subject to environmental variations, but more studies are needed to confirm this relationship. In addition, the *Staphylococcus* genus is also the most prevalent microorganism in eye lesions in horses [22].

Treatments for ulcerative keratitis often have limited results. Therefore, there is a need to find new therapeutic strategies that are of multiple actions, are less artificial, and are without potential allergens, such as preservatives or other products that, in the short or long term, can induce toxicity on the ocular surface, which is very vulnerable [23]. In this context, platelet-rich plasma (PRP), which is a blood-derived biological product, is used to promote better tissue repair, through the growth factors (GFs) contained in its composition, and fulfills most therapeutic goals for this type of eye pathology. Bezerra et al. [24] demonstrated the effectiveness of PRP against ulcerative keratitis in a horse, in which there was a reduction in consolidation time and a better healing quality. In addition to the therapeutic advantages already mentioned, its antimicrobial potential has been receiving attention. In humans, they reported that platelet concentrate has antimicrobial activity against bacteria such as *Staphylococcus aureus* (methicillin-resistant and sensitive), *Escherichia coli*, and others [25, 26]. In horses, the effect of PRP was also confirmed in an in vitro model against methicillin-resistant and sensitive *Staphylococcus aureus* [27, 28]. These studies concluded that the combination of PRP components such as platelets and their alpha granules that release some microbicidal proteins and growth factors, such as leukocytes and plasma that contain complement and complement-binding proteins, produces a substance with antimicrobial effects [27, 28].

This study aims to demonstrate the antimicrobial effect of platelet-rich plasma (PRP), its time of action, and its correlation with the concentration of its same components in vitro on *Staphylococcus sciuri*, a bacterium with high prevalence in the normal ocular microbiota of horse healthy in the municipality of Minas Gerais. To date, no studies have reported the antimicrobial action of PRP on bacteria isolated from the normal equine ocular microbiota, either in vitro or in vivo. For this study, several antimicrobial tests were applied, where one of the objectives of this fact was the search for techniques that could reduce the instability of the CFU variable, a gold standard test to evaluate bacterial growth, for which it was decided to also use spectrophotometry to evaluate the bacterial growth pattern of the different dilutions tested.

If the antimicrobial effect in this situation is confirmed, the use of antimicrobials for eye treatments in horses can be reduced, using an easily obtained biological therapy, with no reported side effects. In addition, this therapy will likely also have advantages in modulating inflammation and tissue repair, as has been observed for other tissues and diseases.

## 2. Materials and Methods

This study was approved by the Ethics Committee on Animal Experimentation of the Federal University of Minas Gerais (CEUA/UFMG) under protocol number 231/2020.

### 2.1. Horses

By means of a complete physical examination and a hematological examination, eight clinically healthy horses (4 males and 4 females) with a mean age of 7 years (range: 4–13) were selected. The animals came from a farm where the environmental and management conditions were the same for everyone, respecting all animal welfare parameters. Horse owners were informed of the nature of the investigation and signed the appropriate authorization.

During the experiment, the animals were not removed from their environment of origin or their routine. Exclusion criteria in the study were changes in initial physical examination, recent use of anti-inflammatory drugs and antibiotics (in the previous 7 days), recent systemic illness, or travel in the last month.

### 2.2. Blood Collection and Preparation of Equine PRP

For blood extraction and PRP processing, a protocol adapted from [29] was used. Aseptic blood collection was performed by venipuncture of the external jugular vein, through the vacuum tube system, with a 22-G vacutainer needle (Agulha, BD Vacutainer, SP, Brazil) and vacuum tubes (Biocon®). In each collection, a total of 37 tubes per animal were extracted, extracting a volume of 130.6 ml of blood per animal. A sample was collected in a 1.0 ml tube of ethylenediaminetetraacetic acid (EDTA) to perform the initial blood count. Then, 36 tubes of 3.6 ml with 3.2% sodium citrate (CIT) were collected per animal, to later make the PRP pool. The samples were homogenized by slow manual inversion of the tubes.

Blood collected in tubes with CIT was processed using a double centrifugation protocol. In the first step, the blood was transferred to sterile 15 ml falcon tubes to optimize processing time and immediately centrifuged (CENTRIBIO 80-2B®, Biovera, Brazil), using 120 g for 5 min with a subsequent rest of 10 min in a vertical position. The first fraction of supernatant plasma (50%) was discarded. The other 50% of plasma was aspirated at 4 mm above the buffy coat with the aid of a graduated pipette of up to 1 ml and a micropipette of 200 *μ*L when it was close to the buffy coat. This fraction was transferred to other sterile 15 ml falcon tubes, and all processing was carried out under aseptic conditions, under a laminar flow hood. After the second centrifugation, performed at 240 g for 5 min with subsequent rest for 10 min in an upright position, 75% of the first fraction was discarded. The remaining 25% was pipetted to dissolve the pellet formed in the final portion of the tube. This last fraction was considered as PRP after the final platelet count above 300,000/*μ*L, as described by [30–32]. Red blood cell and leukocyte counts were also performed using an impedance hemocytometer (Icounter Vet®, DIAGNO, Brazil).

Finally, the PRP of each individual was transferred to a 100 ml polypropylene beaker to obtain a single pool of all animals, which yielded a final amount of 66 ml of PRP. From this pool, another aliquot was separated to obtain platelet concentration, erythrocyte, and leukocyte counts on an impedance hemocytometer.

### 2.3. Study Design and *In Vitro* Antibacterial Assay

A pool of PRP without prior activation was analyzed to determine its antibacterial effect against six isolated strains of *Staphylococcus sciuri* and a standard strain of *Staphylococcus aureus* “American Type Culture Collection (ATCC-29213, OxoidCulti-Loops, KS, USA).”

Due to the possible individual variability of the strains, six strains isolated from different animals of *Staphylococcus sciuri* (S1–S6) were used, the most prevalent microorganism in the ocular microbiota of healthy horses from the metropolitan area of Belo Horizonte-MG (data not yet published) from the bacterial bank of the Veterinary School of UFMG, Laboratory of Bacteriology. In addition to these strains, a standard strain (SS) of *Staphylococcus aureus* ATCC-29213 was included to ensure the reliability and reproducibility of the results.

The minimum inhibitory concentration (MIC) was evaluated by the macrodilution method in tubes with Brain Heart Infusion Broth (BHI) (Brain Heart Infusion Broth ®, KASVI, Brazil) at all times, and samples were visually inspected, according to the standards established by the “National Committee for Clinical Laboratory Standard” (NCCLS).

**Figure 1:**
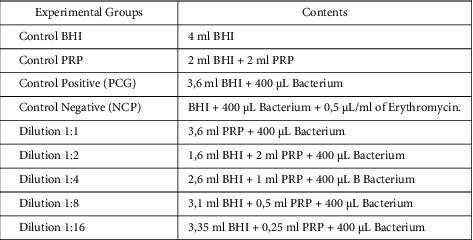
Design of the subgroups of each treatment.

The experimental groups were confirmed for all bacterial strains tested by five dilutions of PRP (1 : 1, 1 : 2, 1 : 4, 1 : 8, 1 : 16) together with BHI broth and 400 *μ*L of bacteria to obtain a concentration end of inoculum of 1 × 10^5^ CFU/ml. For each strain, four control groups were established, the BHI control consisted of only BHI broth, the PRP control consisted of BHI broth and pure PRP, the positive control group (PCG) consisted of BHI broth with bacteria, and a control group negative (NCP) formed by BHI broth with bacteria and 0.5 *μ*L/ml of purified erythromycin for antibiotic sensitivity test (Drogavet®, Brazil), minimum inhibitory dose for the two bacteria tested according to NCCLS. In Figure 1, we can see the composition of each experimental group; all groups had a final volume of 4 ml.

All samples were incubated at 37°C for 24 hours. During this incubation time in the broth, aliquots were extracted for each dilution of PRP (1 : 1, 1 : 2, 1 : 4, 1 : 8, 1 : 16) and the PCG of 100 *μ*L after 3, 6, 12, and 18 hours of incubation for all samples. These aliquots were diluted in phosphate buffer saline solution (PBS 90%) to 10^−^³. Immediately afterward, 100 *μ*L of the sample was seeded on 5% horse blood agar plates (Base Blood Agar ®, KASVI, Brazil). All processing was performed under aseptic conditions in a laminar flow hood. These plates were labeled and incubated at 37°C for 24 hours for subsequent manual counting of CFUs in each plate. The highest growth observed for any sample was limited to 300 CFU/plate for all dilutions. The number of CFU/mL will be determined by the following formula: CFU/mL = CFU/plate × 1/0.1 ml (aliquot factor) × 10³ (dilution factor) [28].

After completion of the 24 hours of culture in the broth for the NCG, a visual reading was carried out first, looking for the formation of bacterial mass or turbidity in the samples, and then soon after, sowing in a Petri dish to evaluate bacterial growth and the effectiveness of the antibiotic in these microorganisms.

At the time of 0 hours, aliquots of the BHI control and PRP control groups were taken for seeding and cultivation in a Petri dish, to validate the sterility of the products.

To test a technique that could reduce the instability of the variable CFU, it was decided to also use spectrophotometry to evaluate the bacterial growth pattern of the different dilutions tested. For the reading of the optical density on the spectrophotometer, during the 24 hours of incubation of the samples, aliquots of 400 *μ*L were extracted at times 0, 3, 6, 12, and 18 hours for each tested dilution of the product. These aliquots were read in a quartz microcuvette, using the spectrophotometer method (Spectrophotometer 35 D®, Coleman, Brazil) in absorbance, with a wavelength of 620 nm, using BHI broth to zero the instrument and then reading the samples.

### 2.4. Statistical Analysis

Statistical analysis was performed using the R software version 3.6.1 (R Core Team, 2019).

In this study, the horse does not constitute the experimental unit, as the individual character will not be considered, mainly because a pool of different PRPs will be carried out. The number of animals used for blood collection was defined based only on the amount of blood needed for all analyses, using a volume much smaller than the maximum allowed per animal (8% of the live weight).

To test the differences between strains, times, and dilutions, linear regression models were fitted. The different strains, times, and dilutions were used as predictor variables. It was necessary to apply the logarithmic transformation to the response variables to control the variance and reduce the impact of outliers. After adjusting the model, the mean values were calculated, and their respective 95% confidence intervals and illustrative graphs were constructed. Multiple comparison tests (pairwise) were performed by applying the Sidak correction. For all tests, a significance level of 5% was used.

## 3. Results

One of the strains (S2) was removed from the CFU/ml and optical density data analysis because it showed bacterial inactivity during the experiment.

### 3.1. Blood Count and Assessment of Whole Blood Compared to PRP

The results of the hemograms of the individuals were within the reference values for the equine species. The mean blood counts were 6.57 × 10^6^ ± 0.38 red blood cells/*μ*L, 11.02 × 10³ ± 2.5 leukocytes/*μ*l, and 187 × 10³ ± 47.4 platelets/*μ*L.

The PRPs of the 8 animals showed mean values of 0.01 × 10^6^ ± 0.002 red blood cells/*μ*L, 2.62 ± 0.80 × 10³ leukocytes/*μ*L, and 344.4 × 10^3^ ± 13.2 platelets/*μ*L.

After measuring the individual PRP values, they were grouped into a pool that was defined as the final product to be tested. The values of this pool were 0.01 × 10^6^ red blood cells/*μ*L, 2.06 × 10^3^ leukocytes/*μ*L, and 382 × 10³ platelets/*μ*L, with a platelet concentration of 2.05 times higher than the baseline concentration.

### 3.2. Strains Behavior and Dilutions Over Time Related to Colony Forming Units (CFU) Counts

The 5 strains of *Staphylococcus sciuri* and the standard strain of *Staphylococcus aureus* selected for the study were analyzed according to their behavior at different times, considering the means of the PRP dilutions (1 : 1, 1 : 2, 1 : 4, 1 : 8, and 1 : 16) for CFU counts.  Table 1 shows the median values and their respective quartiles. The values recorded in the study as >300 × 10^4^ were transformed to 300 × 10^4^.

At time 0, descriptive means were calculated for the samples taken from the BHI control, PRP control, and positive control groups for both *Staphylococcus sciuri* and *Staphylococcus aureus*. For the two microorganisms, the mean values obtained in the BHI control groups and the PRP control groups were 0.0 × 10^4^ CFU/ml and 0.0 × 10^4^ CFU/ml, respectively. These findings reflect sterility in the preparation of the BHI broth. Also, with regards to the PRP control group, it suggests that the product to be tested was not contaminated in its preparation. The positive control group (PCG) presented a value of 8 × 10^4^ CFU/ml, which demonstrates that bacteria were viable and able to replicate.

**Figure 2:**
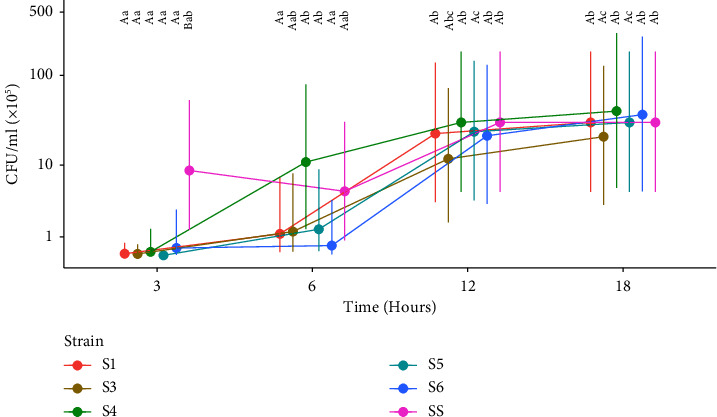
-Means of CFUs/ml of the strains over time, considering all means of dilutions. ^*∗*^S1–6: *Staphylococcus sciuri*; SS: *Staphylococcus aureus*. Different uppercase letters specify significant differences between strains for the same time, and different lowercase letters specify significant differences between times for the same strain, with a significance level of 5%.

Figure 2 shows the linear graph that represents the behavior of the CFU variable of each strain over time.

Comparing the differences between strains for the same time, it was observed that at time 3, SS had a higher bacterial growth (*P* < 0.05) than all other strains of *Staphylococcus sciuri* (Figure 2). Within the other times (6, 12, and 18), there was no difference in bacterial growth between strains, regardless of the dilutions.

Comparing the differences between times for the same strain in (Figure 2), it was observed that strains S1, S3, and S6 showed significant bacterial growth (*P* < 0.05) from time 12 onwards. Strains S4 and S5 started to grow significantly (*P* < 0.05) earlier, at time 6. The S6 strain continued to grow significantly (*P* < 0.05) even after time 6. The SS showed, over time, an unstable growth behavior.

The 5 dilutions tested 1 : 1, 1 : 2, 1 : 4, 1 : 8, and 1 : 16 of our product (PRP) and PCG were also analyzed and compared with each other over time considering all strain means for the CFU scores. In Table 2, we find the median values and their respective quartiles.

**Figure 3:**
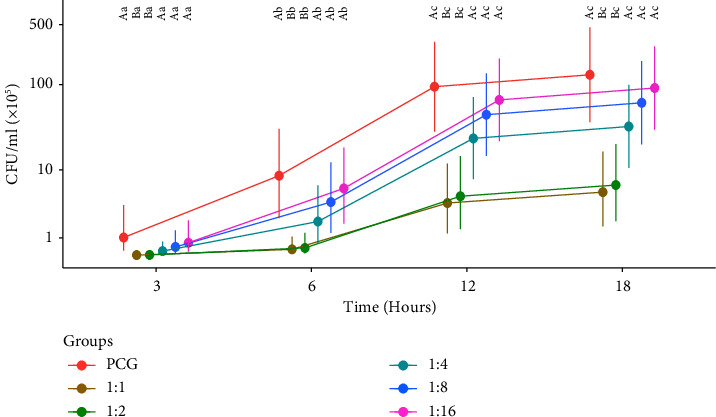
Means of CFU/ml of the dilutions over time, considering the means of the strains. Different uppercase letters specify significant differences between dilutions at the same time. Different lowercase letters specify significant differences between times for the same dilution, with a significance level of 5%. ^*∗*^PCG: positive control group.

**Figure 4:**
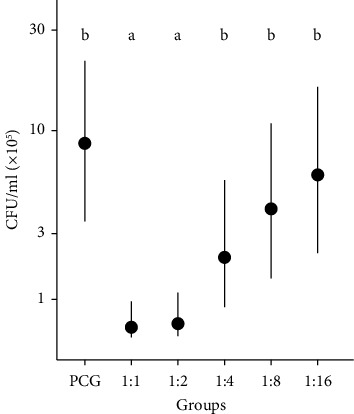
Means of CFU/ml of the dilutions considering all the means of the strains and times grouped. ^*∗*^PCG: positive control group. Different lowercase letters specify significant differences between groups.

In Figure 3, the differences in bacterial growth (CFU/ml) were compared between the tested PRP dilutions and PCG over time. There was less growth of CFUs (*P* < 0.05) and therefore a greater antimicrobial effect in the 1 : 1 and 1 : 2 dilutions when compared to the 1 : 4 dilution onwards and PCG, at all times. This was also observed in Figure 4, considering all unified strain means and time. In turn, when comparing the differences between times for the same dilution (Figure 3), it is observed that all dilutions are observed to show bacterial growth (*P* < 0.05) at times 3, 6, and 12.

### 3.3. Strain Behavior and Dilutions Over Time Related to Optical Density Measured by Absorbance

The 5 strains of *Staphylococcus sciuri* and the standard strain of *Staphylococcus aureus* selected for the study were analyzed according to their behavior at different times. PRP dilutions were tested for absorbance using spectrophotometry at a wave of 620 nm, after which the means were used.  Table 3 presents the descriptive values of the medians and their respective quartiles.

**Figure 5:**
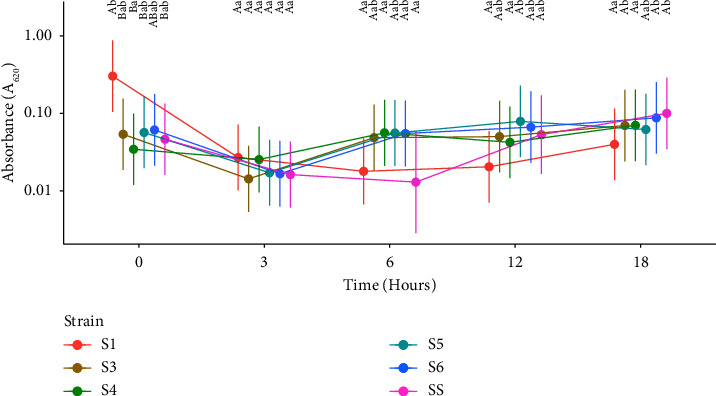
Means of absorbance (*A*_620_) of the strains over time considering all the means of the dilutions. ^*∗*^S1–6: *Staphylococcus sciuri*; SS: *Staphylococcus aureus*. Different uppercase letters specify significant differences between strains at the same time. Different lowercase letters specify significant differences between times for the same strain, with a significance level of 5%.

In Figure 5, the graph represents the absorbance behavior of the strains over time.

Comparing the differences between strains for the same time in Figure 5, we found that S1 showed higher absorbance (*P* < 0.05) than strains S3, S4, S5, and SS at time 0, in any of the dilutions. Within the others, there was no difference between the optical density readings over time.

Comparing the differences between times for the same strain in (Figure 5), regardless of the dilutions, we observed that S1 started reading at time 0 with a higher absorbance, which was reduced (*P* < 0.05) soon after at time 3, staying that way until the end. The increase in absorbance of S3, S6, and SS only became significant (*P* < 0.05) at time 18. At S5, this happened at time 12.

In Table 4, we find the descriptive values of the medians and their respective quartiles of each dilution over time, considering the means of the strains.

**Figure 6:**
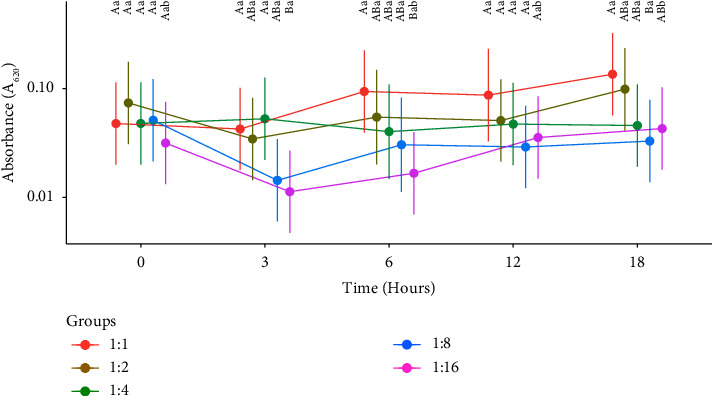
Means of absorbance (*A*_620_) of the dilutions over time considering the means of the strains. Different uppercase letters specify significant differences between dilutions at the same time. Different lowercase letters specify significant differences between times for the same dilution, with a significance level of 5%.

**Figure 7:**
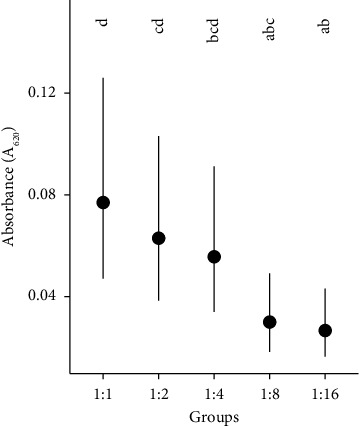
Means of the dilutions absorbance considering all strain means and times grouped. Different lowercase letters specify significant differences between groups.

In Figure 6, when all strains were grouped together, the differences in absorbance were compared between the tested PRP dilutions over time.  Figure 7, both strains and times were grouped.

In Figure 6, comparing the differences between dilutions for the same time, it is observed that the 1 : 1 dilution had higher absorbance (*P* < 0.05) at times 3 and 6 than the 1 : 16 dilution. The 1 : 4 dilution also showed higher absorbance (*P* < 0.05) at time 3 than the 1 : 16 dilution. Furthermore, the 1 : 1 dilution had a significantly (*P* < 0.05) higher absorbance at time 18 than the 1 : 8 dilution. On the other hand, comparing the differences between times for the same dilution, only the 1 : 16 dilution showed lower absorbance at time 3 compared to time 18 (*P* < 0.05).

In Figure 7, it can be seen that the absorbance of the 1 : 1 dilution was greater than that of the 1 : 8 and 1 : 16 dilutions. The 1 : 2 dilution had a significantly (*P* < 0.05) higher optical density (*P* < 0.05) compared to the 1 : 16 dilution.

## 4. Discussion

The use of platelet-rich plasma (PRP) represents a simple form of treatment in equine ophthalmology, which can stimulate an increase in the proliferative and migratory capacity of corneal cells, reducing the time for consolidation and providing better healing in ocular surface injuries [23, 33]. In equine veterinary medicine, few studies have evaluated the antimicrobial effect of blood components. This study is the first to evaluate the antimicrobial effect of PRP against the *Staphylococcus sciuri* microorganism of the ocular microbiota of healthy horses. Until now, equine blood products have shown an antimicrobial effect against methicillin-sensitive *Staphylococcus aureus* (MSSA) and methicillin-resistant *Staphylococcus aureus* (MRSA) [27, 28] and *Escherichia coli* [21].

Our PRP presented a difference, however not high, between the mean platelet values of the individual horses compared to the values found in the pool. This happened due to the fact that the individual factor affected the concentration of the pool, since some animals concentrated more platelets than others.

Although there is no standardization of protocols for the acquisition of ideal cellular components in equine PRP, the product used in this study was classified as a PRP with adequate platelet concentration [28, 30–32] and considered as a pure-platelet-rich plasma (P-PRP) [34] or as leukoreduced platelet concentrate [35], making it a safer product for use in equine ophthalmology, considering that PRP with high concentrations of leukocytes promotes catabolic processes in fabrics [30].

In this study, it was observed that SS (*S. aureus*) had greater growth at 3 hours, which is logical considering that it is a pathogenic agent [36]. The other strains (S1–S6) are strains of the ocular microbiota of healthy animals; therefore, they possibly do not have the same replication and colonization efficiency. It was clear that PRP was effective in inhibiting these bacteria as early as 3 hours. The inhibition of PRP on SS only occurred after 6 hours, which may mean that the inhibitory factors present in PRP needed a longer time to have an effect. From a clinical point of view, this may be particularly important when trying to identify *S. aureus* as the causative agent of ulcerative keratitis in horses [22]. Different results were observed by other researchers, who cultivated PRP and other blood products, with MSSA and MRSA directly on the blood agar plate, and read the plates at 6 and 24 hours, comparing their treatments and the control between them. On reading after 6 hours of cultivation in the case of PRP, it totally inhibited bacterial growth compared to its control group. At 24 hours, the CFU/ml values of PRP were lower than those obtained in the control group [28].

Another interesting and occasional finding in this study was the coagulation of SS samples from all subgroups since time 3. This fact is supported by the literature, since *Staphylococcus aureus* has a protein called coagulase that allows the conversion of fibrinogen to fibrin, interacting with von Willebrand factor binding protein, which binds to prothrombin (factor II of the clotting cascade), forming a complex called staphylothrombin [37] which then uses fibrinogen binding proteins such as aggregation factor (A) to adhere to fibrin clots and form fibrin-containing bacterial aggregates [38]. This fibrin membrane makes the bacteria resistant to phagocytosis through this mechanism [24, 27]. This mechanism clearly demonstrates that there was an interaction of PRP with the bacteria, which could not have been demonstrated in MIC curves with antibiotics. Possibly, this mechanism influenced the greater bacterial growth of SS at time 3 when compared to *Staphylococcus sciuri* strains. It is also possible that the reduction of SS CFUs only at time 6, although not significant, could be attributed to a delay in the effectiveness of the PRP, due to the same mechanisms.

Although the present study also proposed to analyze and define a MIC for PRP using the macrodilution method in BHI broth, this did not prove to be an effective technique for evaluating the effects of PRP in the methodology used. Furthermore, our study aimed to make observations at shorter time intervals and not just after 24 hours of cultivation. Another important factor was the fact that the density of PRP made it difficult to visually evaluate the samples, especially the SS ATCC subgroups of *S. aureus*, due to the coagulation of the samples. No studies were found that used the visual evaluation methodology to define MIC in equine PRP. Thus, it was decided to interpret the results of the dilutions in the CFU counts, which is the gold standard test to evaluate bacterial growth.

In this study, PRP was not activated, but the results showed that there was indeed an inhibitory effect, a finding that generates controversy compared to [39] who stated in their study that blood products without prior activation did not present an inhibitory effect. On the other hand, the authors in [28, 40] showed that activated PRP preparations had lower antimicrobial activity against some microorganisms tested compared to inactivated preparations. These findings are believed to be due to complement consumption during activation of coagulation [41]. Platelet activation was evident in SS since it promoted the clotting of samples after contact with PRP. This event is supported by the literature since platelets have several mechanisms of interaction with bacteria, which causes them to activate [42, 43]. In addition, although *Staphylococcus sciuri* strains 1–6 do not have some virulence factors to generate PRP coagulation, the interaction between product and bacteria was evident from the results obtained, showing inhibition of bacterial growth of these strains when compared to the group positive control.

The PRP in this study showed an inhibition of bacterial growth of all strains tested during the first hours of incubation, but without reaching the complete breakdown of the microbial load, showing a recovery of bacterial growth. With this, it can be indicated that PRP presented a bacteriostatic inhibitory activity instead of a microbicidal one, and these results are compatible with what some authors have said, who also evaluated the potential of PRP and other blood products [26, 28, 44–47].

The time that the PRP tested in this study showed an inhibitory effect on bacterial growth was up to 6 hours for both *Staphylococcus sciuri* and *Staphylococcus aureus*. Other authors reported a time of inhibitory effect of PRP and other blood products against different microorganisms between a range of 4–8 hours, which is compatible with those obtained in this study [26, 28, 44–47]. This inhibitory time can be variable and discrepant due to several factors, including the intrinsic characteristics of the bacterial strains used, which may have different susceptibility to different platelet concentrates and their composition, or to the different sensitivity of the test used to assess antibacterial activity.

Taking into account the results obtained in this study, we can say that the use of PRP can be used either pure 1 : 1 or diluted up to 1 : 2, presenting a bacteriostatic effect of up to 6 hours, against all strains of *Staphylococcus sciuri*, a resident microorganism of the ocular conjunctival microbiota of healthy horses and against *Staphylococcus aureus* (SS), a multidrug-resistant pathogenic microorganism. Therefore, it can also be inferred that the antimicrobial properties of PRP are concentration dependent, which is consistent with the results of [48]. Although this study did not assess which of the cellular components of PRP is responsible for antimicrobial activity, our results support the hypothesis that the in vitro antimicrobial effect can probably be attributed to platelets [49, 50] and plasma complement [28, 39], since our PRP was a leukoreduced blood product. However, it is also possible that the antimicrobial effect of PRP under in vivo conditions could be mediated by other biological mechanisms that include leukocytes or the combination of all these components makes PRP a biological therapy with antimicrobial effects [28, 41, 44].

Although this study has proposed using absorbance as a measure of optical density by spectrophotometry in the search for a faster, more sensitive method with more stable data compared to CFUs. This search was restricted by the stable behavior of the spectrophotometry results, therefore, without following any pattern either positive or negative in bacterial growth and by the fact that PRP, being a blood derivative that contains a mixture of platelets, leukocytes, growth factors, serum, and plasma proteins [27, 39], directly affects the absorbance result, reading all this PRP cellularity and, in addition, the bacteria are alive or dead. In addition, PRP presented in its 1 : 1 and 1 : 2 dilutions greater (*P* < 0.05) absorbance than the other dilutions, a fact that supports that the same concentration of PRP interfered with the results of this test. An interesting finding in this test was the fact that the 1 : 16 dilution, with the lowest concentration of PRP, showed a significant difference in bacterial growth at time 3, time in which it had the lowest absorbance, compared to time 18, when it had its highest absorbance value. This fact demonstrates a tendency for this strain to increase its absorbance due to the same fact that lower concentrations of PRP do not lead to an effective antibacterial effect as in higher concentrations.

## 5. Conclusions

Under the conditions of the present experiment, evaluating the antimicrobial effect of PRP against *Staphylococcus sciuri*, a resident microorganism of the normal conjunctival ocular microbiota of healthy horses in the city of Minas Gerais, the following conclusions can be obtained:

- Strains isolated from the normal conjunctival microbiota of *Staphylococcus sciuri* were more susceptible to the antimicrobial effect of PRP than the ATCC strain of *Staphylococcus aureus*
- PRP showed a bacteriostatic antimicrobial effect in vitro for up to 6 hours, at 1 : 1 and 1 : 2 dilutions
- The antimicrobial effect of PRP is concentration-dependent
- Spectrophotometry is not an applicable method to assess the interaction and behavior of bacteria against PRP

## Figures and Tables

**Table 1:** Medians of CFU/ml with their respective quartiles of strains at each time point, considering all median dilutions.

Strains	Time 3			Time 6			Time 12			Time 18		
	1° quartile	Median	3° quartile	1° quartile	Median	3° quartile	1° quartile	Median	3° quartile	1° quartile	Median	3° quartile
S1	0.0 × 10^4^	0.0 × 10^4^	3.0 × 10^4^	2.0 × 10^4^	3.0 × 10^4^	46 × 10^4^	54 × 10^4^	300 × 10^4^	300 × 10^4^	300 × 10^4^	300 × 10^4^	300 × 10^4^
S3	0.0 × 10^4^	0.0 × 10^4^	1.0 × 10^4^	0.0 × 10^4^	38 × 10^4^	153 × 10^4^	1.0 × 10^4^	300 × 10^4^	300 × 10^4^	239 × 10^4^	300 × 10^4^	300 × 10^4^
S4	0.0 × 10^4^	4.0 × 10⁴	10 × 10^4^	62 × 10^4^	300 × 10⁴	300 × 10^4^	300 × 10^4^	300 × 10^4^	300 × 10^4^	300 × 10^4^	300 × 10^4^	300 × 10^4^
S5	0.0 × 10^4^	0.0 × 10^4^	0.0 × 10^4^	1.0 × 10^4^	14 × 10^4^	70 × 10^4^	136 × 10^4^	300 × 10^4^	300 × 10^4^	300 × 10^4^	300 × 10^4^	300 × 10^4^
S6	0.0 × 10^4^	4.0 × 10^4^	20 × 10^4^	0.0 × 10^4^	1 × 10^4^	300 × 10^4^	147 × 10^4^	300 × 10^4^	300 × 10^4^	300 × 10^4^	300 × 10^4^	300 × 10^4^
SS	104 × 10^4^	117 × 10^4^	154 × 10^4^	3 × 10^4^	88 × 10^4^	300 × 10^4^	300 × 10^4^	300 × 10^4^	300 × 10^4^	300 × 10^4^	300 × 10^4^	300 × 10^4^

S1–6: *Staphylococcus sciuri*; SS: *Staphylococcus aureus*.

**Table 2:** Medians of CFU/ml with their respective quartiles of dilutions according to time considering all strains.

Groups	Time 3			Time 6			Time 12			Time 18		
	1° quartile	Median	3° quartile	1° quartile	Median	3° quartile	1° quartile	Median	3° quartile	1° quartile	Median	3° quartile
PCG	32.5 × 10^4^	38 × 10^4^	110.5 × 10^4^	300 × 10^4^	300 × 10^4^	300 × 10^4^	300 × 10^4^	300 × 10^4^	300 × 10^4^	300 × 10^4^	300 × 10^4^	300 × 10^4^
1 : 1	0.0 × 10^4^	0.0 × 10^4^	0.0 × 10^4^	0.0 × 10^4^	0.0 × 10^4^	2.0 × 10^4^	72 × 10^4^	300 × 10^4^	300 × 10^4^	239 × 10^4^	300 × 10^4^	300 × 10^4^
1 : 2	0.0 × 10^4^	0.0 × 10^4^	0.0 × 10^4^	0.0 × 10^4^	0.0 × 10^4^	3.0 × 10^4^	25 × 10^4^	300 × 10^4^	300 × 10^4^	66 × 10^4^	300 × 10^4^	300 × 10^4^
1 : 4	0.0 × 10^4^	3.0 × 10^4^	4.0 × 10^4^	1.0 × 10^4^	14 × 10^4^	300 × 10^4^	300 × 10^4^	300 × 10^4^	300 × 10^4^	300 × 10^4^	300 × 10^4^	300 × 10^4^
1 : 8	0.0 × 10^4^	1.0 × 10^4^	154 × 10^4^	46 × 10^4^	88 × 10^4^	300 × 10^4^	300 × 10^4^	300 × 10^4^	300 × 10^4^	300 × 10^4^	300 × 10^4^	300 × 10^4^
1 : 16	0.0 × 10^4^	14 × 10^4^	23.0 × 10^4^	300 × 10^4^	300 × 10^4^	300 × 10^4^	300 × 10^4^	300 × 10^4^	300 × 10^4^	300 × 10^4^	300 × 10^4^	300 × 10^4^

PCG: positive group control.

**Table 3:** Medians of absorbance (*A*_620_) with their respective quartiles of strains over time, considering all means of dilutions.

Strains	Time 0			Time 3			Time 6			Time 12			Time 18		
	1° qua	Median	3° qua	1° qua	Median	3° qua	1° qua	Median	3° qua	1° qua	Median	3° qua	1° qua	Median	3° qua
S1	0.2800	0.4300	0.6200	0.0260	0.0310	0.0610	0.0020	0.0170	0.1160	0.0120	0.0195	0.0250	0.0080	0.0685	0.0700
S3	0.0470	0.0635	0.0730	0.0060	0.0240	0.0460	0.0200	0.0720	0.0850	0.0460	0.0625	0.0770	0.0500	0.0765	0.0800
S4	0.0230	0.0575	0.0700	0.0220	0.0320	0.0710	0.0400	0.0700	0.1000	0.0180	0.0510	0.0640	0.0500	0.0725	0.0880
S5	0.0450	0.0575	0.1050	0.0040	0.0300	0.0490	0.0360	0.0600	0.0970	0.0580	0.0950	0.1170	0.0580	0.0860	0.1120
S6	0.0400	0.0890	0.1350	0.0150	0.0530	0.0880	0.0500	0.0530	0.0890	0.0600	0.0765	0.1220	0.0460	0.0870	0.1220
SS	0.0280	0.0600	0.0770	0.0020	0.0280	0.0740	0.0050	0.0070	0.0260	0.0240	0.0390	0.0760	0.0370	0.0835	0.1210

S1–6: *Staphylococcus sciuri*; SS: *Staphylococcus aureus;* Qua: quartile.

**Table 4:** Medians of absorbance (*A*_620_) with their respective quartiles of dilutions over time considering all strains.

Groups	Time 0			Time 3			Time 6			Time 12			Time 18		
	1° qua	Median	3° qua	1° qua	Median	3° qua	1° qua	Median	3° qua	1° qua	Median	3° qua	1° qua	Median	3° qua
1 : 1	0.0700	0.0770	0.1050	0.0460	0.0610	0.1160	0.0850	0.1290	0.1350	0.0730	0.0760	0.1220	0.0880	0.1220	0.1410
1 : 2	0.0230	0.0580	0.1350	0.0240	0.0530	0.1160	0.0700	0.0845	0.1090	0.0250	0.0600	0.1250	0.0780	0.1120	0.1220
1 : 4	0.0470	0.0570	0.0980	0.0430	0.0490	0.0740	0.0410	0.0710	0.0890	0.0240	0.0600	0.0810	0.0240	0.0460	0.0700
1 : 8	0.0280	0.0400	0.0750	0.0070	0.0260	0.0300	0.0400	0.0550	0.0590	0.0140	0.0230	0.0530	0.0270	0.0320	0.0580
1 : 16	0.0110	0.0280	0.0450	0.0100	0.0130	0.0310	0.0050	0.0200	0.0510	0.0150	0.0390	0.0720	0.0170	0.0670	0.0790

^*∗*^Qua: quartile.

## Data Availability

The data used to support the findings of this study may be released upon application to the Escola de Veterinaria-Universidade Federal de Minas Gerais, who can be contacted at luisdsb94@gmail.com.

## References

[1] Alsousou J., Ali M., Willett K., Harrison P. (2013). The role of platelet-rich plasma in tissue regeneration. *Platelets*.

[2] Etulain J., Mena H. A., Meiss R. P. (2018). An optimised protocol for platelet-rich plasma preparation to improve its angiogenic and regenerative properties. *Scientific Reports*.

[3] Tambella A. M., Martin S., Cantalamessa A., Serri E., Attili A. R. (2018). Platelet-rich plasma and other hemocomponents in veterinary regenerative medicine. *Wounds*.

[4] Tambella A. M., Attili A. R., Dini F. (2014). Autologous platelet gel to treat chronic decubital ulcers: a randomized, blind controlled clinical trial in dogs. *Veterinary Surgery: Vysokomolekulyarnykh Soedinenii*.

[5] Tambella A. M., Attili A. R., Dupré G. (2018). Platelet-rich plasma to treat experimentally-induced skin wounds in animals: a systematic review and meta-analysis. *PLoS One*.

[6] Huang Y., Liu X., Xu X., Liu J. (2019). Intra-articular injections of platelet-rich plasma, hyaluronic acid or corticosteroids for knee osteoarthritis: a prospective randomized controlled study. *Orthopäde, Der*.

[7] Faillace V., Tambella A. M., Fratini M., Paggi E., Dini F., Laus F. (2017). Use of autologous platelet-rich plasma for a delayed consolidation of a tibial fracture in a young donkey. *Journal of Veterinary Medical Science*.

[8] Del Fabbro M., Bucchi C., Lolato A., Corbella S., Testori T., Taschieri S. (2017). Healing of postextraction sockets preserved with autologous platelet concentrates. A systematic review and meta-analysis. *Journal of Oral and Maxillofacial Surgery*.

[9] Tambella A. M., Bartocetti F., Rossi G. (2020). Effects of autologous platelet-rich fibrin in post-extraction alveolar sockets: a randomized, controlled split-mouth trial in dogs with spontaneous periodontal disease. *Animals*.

[10] Alio J. L., Rodriguez A. E., De Arriba P., Gisbert S., Abdelghany A. A. (2018). Treatment with platelet-rich plasma of surgically related dormant corneal ulcers. *European Journal of Ophthalmology*.

[11] Li G., Yin J., Ding H., Jia W., Zhang C. (2013). Efficacy of leukocyte- and platelet-rich plasma gel (L-PRP gel) in treating osteomyelitis in a rabbit model. *Journal of Orthopaedic Research*.

[12] Gilbertie J. M., Schaer T. P., Schubert A. G. (2020). Platelet-rich plasma lysate displays antibiofilm properties and restores antimicrobial activity against synovial fluid biofilms in vitro. *Journal of Orthopaedic Research*.

[13] Attili A. R., Iacoucci C., Serri E. (2021). Antibacterial properties of canine platelet-rich plasma and other non-transfusional hemo-components: an in vitro study. *Frontiers in Veterinary Science*.

[14] Cárdenas M. L., Caceres Y. N. (2017). Plasma rico en plaquetas: una alternativa terapéutica versátil en enfermedades oftálmicas. *Medicentro (Villa Clara)*.

[15] Khosravi A. R., Nikaein D., Sharifzadeh A., Gharagozlou F. (2014). Ocular fungal flora from healthy horses in Iran. *Journal de Mycologie Medicale*.

[16] Andrew S. E., Nguyen A., Jones G. L., Brooks D. E. (2003). Seasonal effects on the aerobic bacterial and fungal conjunctival flora of normal thoroughbred brood mares in Florida. *Veterinary Ophthalmology*.

[17] Araghi-Sooreh A., Navidi M., Razi M. (2014). Conjunctival bacterial and fungal isolates in clinically healthy working horses in Iran. *Kafkas Univ Vet Fak Derg*.

[18] Johns I. C., Baxter K., Booler H., Hicks C., Menzies-Gow N. (2011). Conjunctival bacterial and fungal flora in healthy horses in the UK. *Veterinary Ophthalmology*.

[19] Baran V. I., Özaydin I., Genç O. (2015). The effects of high and low altitudes on conjunctival flora in sport and work horses: a field study in the northeast anatolia region of Turkey (kars and iğdır). *Kafkas Univ. Vet. Fak. Derg*.

[20] Ferreira A. R., Santana A. F., Almeida A. C., Sousa R. F., Perecmanis S., Galera P. D. (2017). Bacterial culture and antibiotic sensitivity from the ocular conjunctiva of horses. *Ciência Rural*.

[21] Hampson E. C., Gibson J. S., Barot M., Shapter F. M., Greer R. M. (2018). Identification of bacteria and fungi sampled from the conjunctival surface of normal horses in South-East Queensland, Australia. *Veterinary Ophthalmology*.

[22] Vercruysse E. M., Narinx F. P., Rives A. C., Sauvage A. C., Grauwels M. F., Monclin S. J. (2022). Equine ulcerative keratitis in Belgium: associated bacterial isolates and in vitro antimicrobial resistance in 200 eyes. *Veterinary Ophthalmology*.

[23] Ribeiro M. V., Melo V. F., Barbosa M. E. (2017). The use of platelet rich-plasma in ophthalmology: a literature review. *Revista Brasileira de Oftalmologia*.

[24] Bezerra N., Moura L., Vago B. (2020). Uso do plasma rico em plaquetas associado a colírios no tratamento de ceratite ulcerativa em equino. *Ciência Animal*.

[25] Bielecki T. M., Gazdzik T. S., Arendt J., Szczepanski T., Kròl W., Wielkoszynski T. (2007). Antibacterial effect of autologous platelet gel enriched with growth factors and other active substances: an in-vitro study. *Journal of Bone & Joint Surgery, British Volume*.

[26] Moojen D. J., Everts P. A., Schure R. M. (2008). Antimicrobial activity of platelet-leukocyte gel againstStaphylococcus aureus. *Journal of Orthopaedic Research*.

[27] Álvarez M. E., López C., Giraldo C. E., Samudio I., Carmona J. (2011). In vitro bactericidal activity of equine platelet concentrates, platelet poor plasma, and plasma against methicillin-resistant *Staphylococcus aureus*. *Archivos de Medicina Veterinaria*.

[28] López C., Álvarez M. E., Carmona J. U. (2014). Temporal bacteriostatic effect and growth factor loss in equine platelet components and plasma cultured with methicillin-sensitive and methicillin-resistant *Staphylococcus aureus*: a comparative in-vitro study. *Veterinary Medicine International*.

[29] Argüelles D., Carmona J. U., Pastor J. (2006). Evaluation of single and double centrifugation tube methods for concentrating equine platelets. *Research in Veterinary Science*.

[30] Fontenot R. L., Sink C. A., Werre S. R., Weinstein N. M., Dahlgren L. A. (2012). Simple tube centrifugation for processing platelet-rich plasma in the horse. *Canadian Veterinary Journal*.

[31] Carmona J. U., López C., Giraldo C. E. (2011). Uso de concentrados autólogos de plaquetas como terapia regenerativa de enfermedades crónicas del aparato musculoesquelético equino. *Archivos de Medicina Veterinaria*.

[32] Yamada A. L., Carvalho A. M., Oliveira P. G. (2012). Plasma rico em plaquetas no tratamento de lesões condrais articulares induzidas experimentalmente em equinos: avaliação clínica, macroscópica, histológica e histoquímica. *Arquivo Brasileiro de Medicina Veterinária e Zootecnia*.

[33] Rushton J. O., Kammergruber E., Tichy A., Egerbacher M., Nell B., Gabner S. (2018). Effects of three blood derived products on equine corneal cells, an in-vitro study. *Equine Veterinary Journal*.

[34] Carmona J. U., López C., Sandoval A. (2013). Review of the currently available systems to obtain platelet related products to treat equine musculoskeletal injuries. *Recent Patents on Regenerative Medicine*.

[35] Giraldo C. E., López C., Álvarez M. E., Samudio I. J., Prades M., Carmona J. U. (2013). Effects of the breed, sex and age on cellular content and growth factor release from equine pure-platelet rich plasma and pure-platelet rich gel. *BMC Veterinary Research*.

[36] Cheung G. Y., Bae J. S., Otto M. (2021). Pathogenicity and virulence of *Staphylococcus aureus*. *Virulence*.

[37] Cheng A. G., Mcadow M., Kim H. K., Bae T., Missiakas D. M., Schneewind O. (2010). Contribution of coagulases towards *Staphylococcus aureus* disease and protective immunity. *PLoS Pathogens*.

[38] Mcadow M., Kim H. K., Dedent A. C., Hendrickx A. P., Schneewind O., Missiakas D. M. (2011). Preventing *Staphylococcus aureus* sepsis through the inhibition of its agglutination in blood. *PLoS Pathogens*.

[39] Drago L., Bortolin M., Vassena C., Romanò C. L., Taschieri S., Del Fabbro M. (2014). Plasma components and platelet activation are essential for the antimicrobial properties of autologous platelet-rich plasma: an in-vitrostudy. *PLoS One*.

[40] Wu X., Ren J., Yuan Y., Luan J., Yao G., Li J. (2013). Antimicrobial properties of single-donor-derived, platelet-leukocyte fibrin for fistula occlusion: an in-vitro study. *Platelets*.

[41] Burnouf T., Chou M. L., Wu Y. W., Su C. Y., Lee L. W. (2013). Antimicrobial activity of platelet (PLT)-poor plasma, PLT-rich plasma, PLT gel, and solvent/detergent-treated PLT lysate biomaterials against wound bacteria. *Transfusion*.

[42] Cox D., Kerrigan S. W., Watson S. P. (2011). Platelets and the innate immune system: mechanisms of bacterial-induced platelet activation. *Journal of Thrombosis and Haemostasis*.

[43] Hamzeh-cognasse H., Damien P., Chabert A., Pozzetto B., Cognasse F., Garraud O. (2015). Platelets and infections-complex interactions with bacteria. *Frontiers in Immunology*.

[44] Anitua E., Alonso R., Girbau C., Aguirre J. J., Muruzabal F., Orive G. (2012). Antibacterial effect of plasma rich in growth factors (PRGF®- Endoret®) against *Staphylococcus aureus* and Staphylococcus epidermidis strains. *Clinical and Experimental Dermatology*.

[45] Mariani E., Filardo G., Canella V. (2014). Platelet-rich plasma affects bacterial growth in-vitro. *Cytotherapy*.

[46] Intravia J., Allen D. A., Durant T. J. (2014). In-vitro evaluation of the antibacterial effect of two preparations of platelet rich plasma compared with cefazolin and whole blood. *Muscles Ligaments Tendons Journal*.

[47] Varshney S., Dwivedi A., Pandey V. (2019). Antimicrobial effects of various platelet rich concentrates-vibes from in-vitro studies-a systematic review. *Journal of Oral Biology and Craniofacial Research*.

[48] Segabinazzi L. G., Canisso I. F., Podico G. (2021). Intrauterine blood plasma platelet-therapy mitigates persistent breeding-induced endometritis, reduces uterine infections, and improves embryo recovery in Mares. *Antibiotics*.

[49] Aktan Í., Dunkel B., Cunningham F. M. (2013). Equine platelets inhibit *E. coli* growth and can be activated by bacterial lipopolysaccharide and lipoteichoic acid although superoxide anion production does not occur and platelet activation is not associated with enhanced production by neutrophils. *Veterinary Immunology and Immunopathology*.

[50] Blair P., Flaumenhaft R. (2009). Platelet alpha-granules: basic biology and clinical correlates. *Blood Reviews*.

